# DOTATATE Scan in Meningiomatosis in a Patient With Ophthalmoplegia

**DOI:** 10.1155/crop/9978785

**Published:** 2026-01-04

**Authors:** Joshua Pasol, Carolina G. Benjamin, W. David Honeycutt

**Affiliations:** ^1^ Bascom Palmer Eye Institute Department of Ophthalmology, University of Miami Miller School of Medicine, Plantation, Florida, USA, miami.edu; ^2^ Department of Neurological Surgery, University of Miami Miller School of Medicine, Miami, Florida, USA, miami.edu; ^3^ Department of Neurology, Neurology Associates, Maitland, Florida, USA

**Keywords:** cranial nerve palsies, diplopia, DOTATATE scan, meningiomatosis

## Abstract

DOTATATE (1,4,7,10‐tetraazacyclododecane‐1,4,7,10‐tetraacetic acid [DOTA]‐octreotate) is a compound that binds somatostatin receptors seen in tumors such as meningiomas. Here, we present a case of a patient with progressive bilateral ophthalmoplegia due to meningiomatosis involving both cavernous sinuses, which was highlighted by DOTATATE imaging. Some studies have shown DOTATATE imaging is superior to contrast‐enhanced magnetic resonance imaging (MRI) in identifying extent of intraosseous meningioma growth, which has been associated with worse prognosis. DOTATATE imaging should be considered in cases of unknown diagnosis of mass lesions and lesions that are in areas not easily accessible to biopsy, such as the cavernous sinuses.

## 1. Case Presentation

A 51‐year‐old female presented with diplopia and progressive ophthalmoplegia since Age 47. She had a past ocular history of left eye amblyopia, accommodative esotropia status–post strabismus surgery performed at Age 8, and hyperopia. At Age 18, she experienced painful diplopia and left facial swelling; she was treated with steroids and diagnosed with Tolosa–Hunt syndrome (THS). At Age 28, she had a recurrence of pain in the left forehead, left sided ptosis, and diplopia, which was treated with steroids for presumed THS. At that time, she was found to have incidental meningiomas on magnetic resonance imaging (MRI), which were monitored. Two lesions were documented on MRI in 2014, a right orbital roof and a planum lesion. She was followed by her strabismologist who noted progressive vertical gaze palsy, unreactive pupils, and a larger angle exotropia, which led to neuro‐ophthalmic referral.

The eye examination showed a BCVA of 20/20 OD and 20/30 OS. Humphrey visual fields 24‐2 were normal. The anterior segment exam showed punctate epithelial erosions OU, whereas posterior exam showed nasal drusen of the right optic disc and retinal pigmentary epithelial changes in the macula OU. The external exam showed 2 mm left upper lid ptosis. Pupils measured 5 mm OD and 5.5 mm OS in the light and 5.5 mm OD and 6 mm OS in the dim room. Both pupils were poorly reactive to light. The ocular motility showed 50% supraduction OD in the direction of the superior rectus and inferior oblique and 25% limitation in adduction consistent with a partial right third nerve palsy. The left eye had 50% limitation in supraduction and adduction, 100% limitation in the direction of the inferior rectus and 25% limitation in abduction consistent with left third and sixth nerve palsies. Retinal nerve fiber layer (RNFL) optical coherence tomography (OCT) measured an average thickness of 82 *μ*m OD and 92 *μ*m OS, whereas the ganglion cell OCT measured 84 and 83 *μ*m, respectively, suggestive of mild optic neuropathy OD.

**Figure 1:**
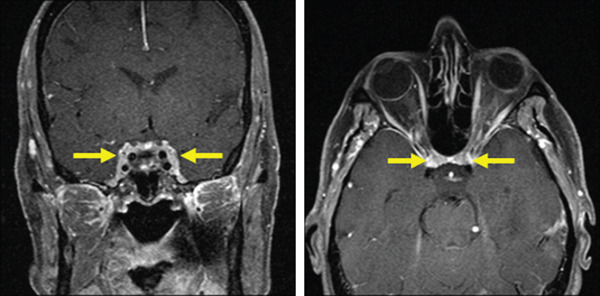
On the left panel is a coronal post‐gadolinium T1 magnetic resonance imaging (MRI) showing abnormal enhancement of both cavernous sinuses (bold arrows) as seen in meningiomatosis. On the right is the axial T1 post‐gadolinium showing bilateral peri‐optic canal meningiomas (bold arrows) straddling both optic nerves not previously reported on prior imaging.

**Figure 2:**
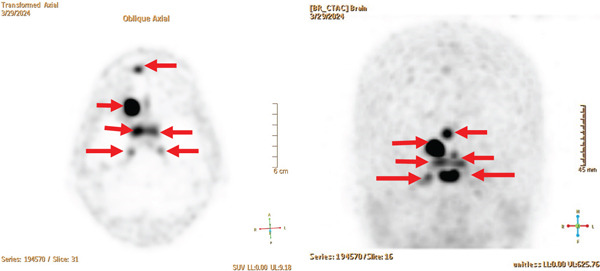
Transformed Cu‐64 DOTATATE PET/CT scan images on this patient with meningiomatosis. The dark areas with arrows represent meningiomas (left, axial section and right, coronal section), which contain somatostatin receptors that bind the tracer used in the DOTATATE scan as seen here. Less intense lesions may signify smaller size or less somatostatin receptors.

MRI images were reviewed, which showed bilateral cavernous sinus enhancement, bilateral peri‐optic canal enhancement (Figure 1), and the previously reported suspected meningiomas consistent with meningiomatosis. The patient was diagnosed with bilateral cavernous sinus meningiomas causing ophthalmoplegia. A previous lumbar puncture was negative for malignancy with only a mildly elevated cerebrospinal fluid protein at 56. Lab testing revealed normal angiotensin converting enzyme (ACE) level, IgG 4, ANCA, RPR, and myasthenia antibodies. The patient was referred for neurosurgical evaluation for treatment of symptomatic meningiomas. A Cu‐64 DOTATATE PET/CT scan was performed, which confirmed multiple meningiomas (Figure 2) including the peri‐optic nerve canals and both cavernous sinuses. The plan was to observe the meningiomas for growth and refer back to her strabismologist for the ocular misalignment. The DOTATATE scan was useful in identifying prior missed meningiomas and aid the diagnosis given prior complicated history of strabismus and THS.

## 2. Discussion

DOTATATE (1,4,7,10‐tetraazacyclododecane‐1,4,7,10‐tetraacetic acid [DOTA]‐octreotate) is a compound that binds somatostatin receptors seen in tumors, such as meningiomas, neuroendocrine tumors, pheochromocytomas, paragangliomas, and oncogenic osteomalacia [1]. DOTATATE is compounded with Gallium‐68, Copper‐64, or Lutetium‐177 to obtain PET/CT imaging for somatostatin receptor tumor location. There are five subtypes of somatostatin receptors, of which somatostatin receptor 2A (SSTR2A) is one of the most commonly expressed in meningioma [2]. DOTATATE scans are used to evaluate meningioma volume for radiation treatment, diagnosing small meningiomas, monitoring tumor growth, and in tumor recurrence in cases where MRI is not clear [3]. A study by Kunz et al. demonstrated that DOTATATE was more sensitive than contrast‐enhanced MRI in the detection of intraosseous meningioma, which is felt to be an important factor in the determination of tumor recurrence and patient mortality [4]. In our case, DOTATATE imaging identified lesions not previously reported in MRI imaging, which helped explain the etiology of the patient′s ophthalmoparesis. Although MRI is the test of choice in evaluating meningioma, DOTATATE imaging can help identify small tumors or aid if the diagnosis is in question such as in optic nerve sheath or en plaque lesions [5]. Another imaging option to consider in assessing meningioma is the use of 68Ga‐DOTATATE PET in combination with MRI. Ivanidze et al. used this type of imaging modality in 20 patients with prior meningioma and found it helped in distinguishing recurrent meningioma from adjacent areas of posttreatment, helped in identifying bone invasion, and confirmed suspected meningiomas as well as recognizing additional meningiomas not previously identified on contrast‐enhanced MRI [6]. If one can identify DOTATATE avidity when there is suspicion of meningioma, one may potentially forgo biopsy if management would not be altered by obtaining a histopathological diagnosis, especially in surgically difficult areas to access such as the cavernous sinuses. In our patient, the plan was to observe the meningiomas and consider radiation to the cavernous sinus lesions and surgery if needed if there is clinical evidence of visual loss. In summary, we can include using DOTATATE imaging to help differentiate meningioma from other non‐somatostatin receptor sensitive processes that cause diplopia or optic neuropathy.

## Ethics Statement

As this case report contains no patient identifier, ethical review board approval is not applicable.

## Consent

No written consent has been obtained from the patients as there is no patient identifiable data included in this case report/series.

## Conflicts of Interest

The authors declare no conflicts of interest.

## Funding

No funding was received for this manuscript.

## Data Availability

Data sharing is not applicable to this article as no new data were created or analyzed in this study.

## References

[1] Hofman M. S. , Lau W. F. , and Hicks R. J. , Somatostatin Receptor Imaging With 68Ga DOTATATE PET/CT: Clinical Utility, Normal Patterns, Pearls, and Pitfalls in Interpretation, Radiographics. (2015) 35, no. 2, 500–516, 10.1148/rg.352140164, 2-s2.0-84924744833, 25763733. 25763733

[2] Schulz S. , Pauli S. U. , Schulz S. , Händel M. , Dietzmann K. , Firsching R. , and Höllt V. , Immunohistochemical Determination of Five Somatostatin Receptors in Meningioma Reveals Frequent Overexpression of Somatostatin Receptor Subtype sst2A, Clinical Cancer Research. (2000) 6, no. 5, 1865–1874, 10815909. 10815909

[3] Wu W. , Zhou Y. , Wang Y. , Liu L. , Lou J. , Deng Y. , Zhao P. , and Shao A. , Clinical Significance of Somatostatin Receptor (SSTR) 2 in Meningioma, Frontiers in Oncology. (2020) 10, 10.3389/fonc.2020.01633, 33014821. PMC7494964 33014821

[4] Kunz W. G. , Jungblut L. M. , Kazmierczak P. M. , Vettermann F. J. , Bollenbacher A. , Tonn J. C. , Schichor C. , Rominger A. , Albert N. L. , Bartenstein P. , Reiser M. F. , and Cyran C. C. , Improved Detection of Transosseous Meningiomas Using68Ga-DOTATATE PET/CT Compared With Contrast-Enhanced MRI, Journal of Nuclear Medicine. (2017) 58, no. 10, 1580–1587, 10.2967/jnumed.117.191932, 2-s2.0-85030844080, 28450556. 28450556

[5] Klingenstein A. , Haug A. R. , Miller C. , and Hintschich C. , Ga-68-DOTA-TATE PET/CT for Discrimination of Tumors of the Optic Pathway, Orbit. (2015) 34, no. 1, 16–22, 10.3109/01676830.2014.959185, 2-s2.0-84925248268, 25264824. 25264824

[6] Ivanidze J. , Roytman M. , Lin E. , Magge R. S. , Pisapia D. J. , Liechty B. , Karakatsanis N. , Ramakrishna R. , Knisely J. , Schwartz T. H. , Osborne J. R. , and Pannullo S. C. , Gallium-68 DOTATATE PET in the Evaluation of Intracranial Meningiomas, Journal of Neuroimaging. (2019) 29, no. 5, 650–656, 10.1111/jon.12632, 2-s2.0-85066087219, 31107591. 31107591

